# Electronic Band Structure and Sub-band-gap Absorption of Nitrogen Hyperdoped Silicon

**DOI:** 10.1038/srep10513

**Published:** 2015-05-27

**Authors:** Zhen Zhu, Hezhu Shao, Xiao Dong, Ning Li, Bo-Yuan Ning, Xi-Jing Ning, Li Zhao, Jun Zhuang

**Affiliations:** 1 Shanghai Ultra-Precision Optical Manufacturing Engineering Center, Department of Optical Science and Engineering, Fudan University, Shanghai 200433, China; 2 State Key Laboratory of Surface Physics and Department of Physics, Fudan University, Shanghai 200433, China; 3 Applied Ion Beam Physics Laboratory, Institute of Modern Physics, Department of Nuclear Science and Technology, Fudan University, Shanghai 200433, China

## Abstract

We investigated the atomic geometry, electronic band structure, and optical absorption of nitrogen hyperdoped silicon based on first-principles calculations. The results show that all the paired nitrogen defects we studied do not introduce intermediate band, while most of single nitrogen defects can introduce intermediate band in the gap. Considering the stability of the single defects and the rapid resolidification following the laser melting process in our sample preparation method, we conclude that the substitutional nitrogen defect, whose fraction was tiny and could be neglected before, should have considerable fraction in the hyperdoped silicon and results in the visible sub-band-gap absorption as observed in the experiment. Furthermore, our calculations show that the substitutional nitrogen defect has good stability, which could be one of the reasons why the sub-band-gap absorptance remains almost unchanged after annealing.

Hyperdoped materials have aroused great interest for the novel electrical and optical properties introduced by the over-saturated impurities. Insulator-to-metal (IMT) transition was observed in S, Se, and Ti hyperdoped silicon^1^,^2^,^3^,^4^, and carrier lifetime recovery with increasing doping concentration was found in Ti hyperdoped silicon^5^. Certainly, the most well-known properties are the significant enhancement of photocurrent generation and broadband absorption of silicon hyperdoped with chalcogens (S, Se, Te)^6^,^7^,^8^,^9^,^10^,^11^,^12^,^13^,^14^,^15^,^16^. More recently, room-temperature sub-band-gap optoelectronic response has also been achieved in silicon by Au hyperdoping^17^. These properties make hyperdoped silicon a promising material in photovoltaic and photoelectricity field.

To obtain the hyperdoped silicon, which has a stable infrared light absorption after annealing and meanwhile has good crystalline quality, we recently prepared the nitrogen hyperdoped silicon by femtosecond pulse laser irradiation in background gas of NF3^18^,^19^. In the sample, the nitrogen peak concentration successfully reached 10^20^ cm^−3^, much higher than the solid solubility limit ~4.5 × 10^15^ cm^−3^. As nitrogen is known to reduce and suppress various defects in silicon^20^,^21^, the NF_3_-prepared sample shows a good crystallinity. In addition, it has a sub-band-gap absorption from near- to mid-infrared wavelength range, which is absent in crystalline silicon. What is more important, the absorptance of the sample remains almost unchanged after annealing^18^,^19^, i.e., it has good thermal stability.

In the present work, to clarify the origin of the sub-band-gap absorption and the good thermal stability, we use first-principles calculations to investigate geometry, electronic band and optical absorption of nitrogen hyperdoped silicon. In the common doped sample, where nitrogen concentration is far below the solubility limit, the structure and diffusion path of nitrogen in silicon were studied extensively for its special effects, such as suppression of void formation, locking dislocation, etc.^20^,^21^,^22^,^23^,^24^. However, for nitrogen hyperdoped silicon, the electronic structure and light absorption are rarely investigated. When the nitrogen concentration greatly exceeds the solubility limit, the impurity band (IB) instead of single impurity level could appear, which means the details of electronic band structure are the key for understanding the unique properties of the hyperdoped sample.

## Results

### Structures of nitrogen defects in silicon

**Figure 1:**
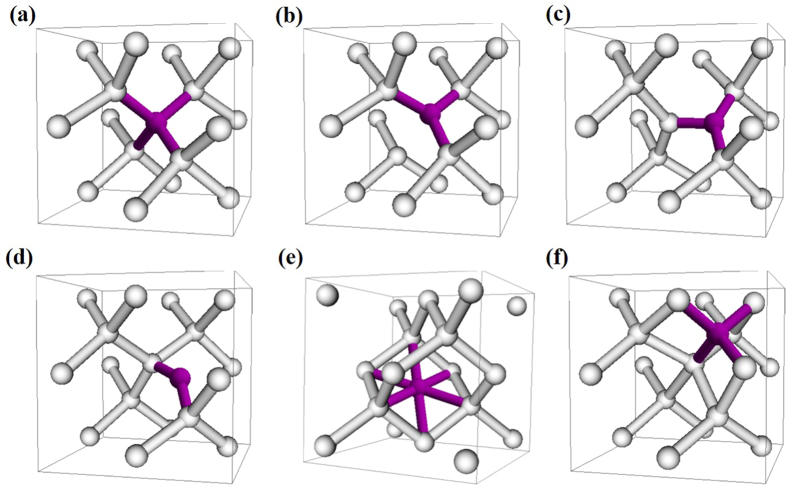
Schematic representation of the defects containing single N atom. White and purple balls represent Si and N atoms, respectively. (**a**) Substitutional structure, N_S_, with on-center nitrogen, (**b**) N_S_ with off-center nitrogen, (**c**) split interstitial structure, N_I_, (**d**) bond-bridge structure, N_B_, (**e**) nitrogen at hexagonal site, N_H_, and (**f**) nitrogen at tetrahedron site, N_T_.

We used the density functional theory (DFT) in the generalized gradient approximation (GGA) with the Perdew-Burke-Ernzerhof (PBE) functional^25^ and the hybrid functional proposed by Heyd *et al.* (HSE06)^26^,^27^ to investigate the atomic geometry, electronic band structure, and optical absorption. The details can be found in the Methods section. For the atomic geometry, we consider two classes of defects, i.e., single and paired nitrogen defects. For single nitrogen, six defect structures were studied as shown in Fig. 1, in which nitrogen could occupy the substitutional or interstitial sites in silicon. Figure 1a,b show substitutional structures denoted as on-center and off-center N_S_ respectively, the difference of which is the specific position of nitrogen. In the on-center N_S_ the nitrogen locates at the normal substitutional site, while in the off-center N_S_ the nitrogen is distorted in <111> direction. It has been found that the off-center N_S_ is more stable than the on-center one^20^,^28^. According to our calculations, the off-center nitrogen with minimal energy locates at 0.57 Å displacement in <111> direction relative to the on-center position, and the energy of off-center nitrogen is lower than that of on-center nitrogen by 0.05 eV (see Table 1), which is close to the experiment value of 0.07 eV^28^. Owing to such small energy difference between off-center and on-center nitrogen, the proportion of on-center nitrogen should be comparable to that of off-center nitrogen, as shown in experiment^28^.

The lower energy structures of interstitial nitrogen have been found to be the split interstitial and bond-bridge geometries as shown in Fig. 1c,d^20^,^21^,^29^. We denoted them as N_I_ and N_B_, respectively. From our calculations, the three N-Si bond-lengths of N_I_ are 1.73, 1.78, and 1.78 Å, the bond along <010> being the shortest. And for N_B_ the two N-Si bond-lengths both are 1.66 Å. These results are consistent with previous calculations^21^. The energy of N_I_ is lower than that of N_B_ by only 0.30 eV, in agreement with the values ≤0.5 eV in references [21,24,29]. The energy difference between N_I_ and N_B_ approximately reflects the diffusion barrier of interstitial nitrogen^24^,^29^. Then this small value 0.30 eV means that the interstitial nitrogen has high diffusion constant in room temperature, and it can easily combine with lattice vacancy to form the N_S_ structure, or combine with another nitrogen atom to form a paired nitrogen structure, etc. This mechanism makes the proportion of interstitial nitrogen to be negligible in silicon^24^,^29^. In contrast, N_S_ structure is stable due to the high binding energy of nitrogen atom with vacancy^20^. The energies are 3.40 and 3.35 eV for off- and on-center substitutional nitrogens respectively, according to the equation described in reference [20]:


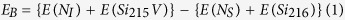


where Si_215_V consists of 215 Si atoms and one vacancy.

The other two interstitial structures, in which nitrogen is at the hexagonal and tetrahedral sites as shown in Fig. 1e,f respectively, have higher formation energies. As shown in Table 1, their energies are higher than that of N_I_ structure by 2.37 eV and 3.0 eV, respectively. Owing to such high energies the structures can be discounted, and will not be discussed in the following.

**Figure 2:**
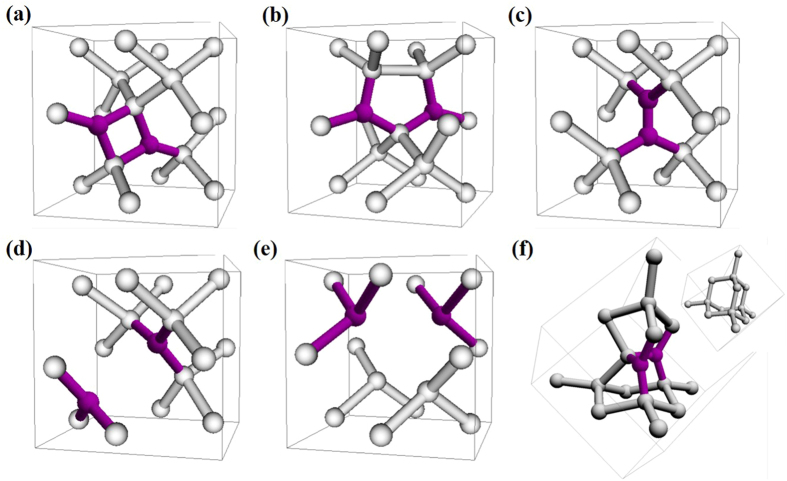
Schematic representation of paired N defect structures. White and purple balls represent the Si and N atoms, respectively. (**a**) The N_I_-N_I_ structure, (**b**) the Humble ring structure, (**c**) the N_I_-N_S_ structure, formed with one substitutional nitrogen and one interstitial nitrogen, (**d**) the N_S_-N_S_ structure, (**e**) the N_S_-N_S_-V structure, formed with two substitutional nitrogens and a vacancy, and (**f**) the N_I_-N_I_-I structure, formed with two interstitial nitrogens and a self-interstitial silicon. The inset of (**f**) is a fraction of defect-free structure for comparison.

There are various possible structures of paired nitrogen in silicon. According to the previous works^20^,^21^, the six main geometries were considered in our calculations. They are N_I_-N_I_, Humble ring, N_I_-N_S,_ N_S_-N_S_, N_S_-N_S_-V and N_I_-N_I_-I structures as shown in Fig. 2. N_I_-N_I_ structure, in which two interstitial nitrogen atoms form a square with two silicon atoms, was thought to be the dominant structure in the nitrogen doped silicon prepared by general methods, such as Czochralski-grown process in nitrogen ambient^21^,^22^. In Humble ring structure, two nitrogen atoms locate at split interstitial sites that are nearest to each other in <110> direction. Its energy is higher than that of N_I_-N_I_ by 0.94 eV per nitrogen. N_I_-N_S_ structure is formed with one substitutional nitrogen and one interstitial nitrogen, and it can be regarded as the combination of a nitrogen pair with a vacancy. N_S_-N_S_ structure has two substitutional nitrogen atoms, and the distance between two substitutional nitrogens is 3.60 Å, which is close to the 3.51 Å obtained by Goss *et al.*^21^. N_S_-N_S_-V represents for the structures formed from the interaction between nitrogen pair and several vacancies. And N_I_-N_I_-I is the structure composed of an interstitial nitrogen pair with a self-interstitial silicon. It was thought that N_I_-N_I_-I appears in the reduction of swirl defects^21^. The structure with lowest formation energy is N_I_-N_I_, which is in agreement with previous calculations^20^,^21^. All the relative formation energies of the paired nitrogen structures are shown in Table 1.

### Electronic band structure of nitrogen hyperdoped silicon

**Figure 3:**
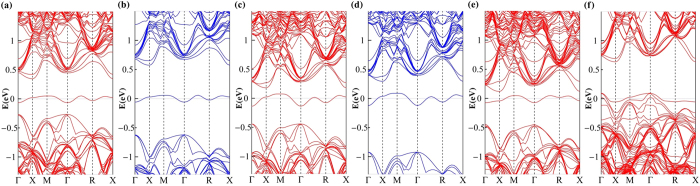
Band structures of single nitrogen defects calculated from the PBE and HSE06 functionals. (**a**) N_S_ with off-center configuration (PBE), (**b**) N_S_ with off-center configuration (HSE06), (**c**) N_S_ with on-center configuration (PBE), (**d**) N_S_ with on-center configuration (HSE06), (**e**) N_I_ (PBE), and (**f**) N_B_ (PBE). The line at zero energy represents the Fermi level.

We performed the calculations for the electronic band structures of nitrogen defects aforementioned. With the PBE functional, the band structures we obtained for the single nitrogen defects are shown in Fig. 3a,c,e,f. We can see that the extra band arising from nitrogen defects could appear in the band gap. For off-center N_S_, whose band structure is shown in Fig. 3a, the extra band is deep into the gap and localized weakly on the N atom (12%), which agrees to the experiment value 9%. And the p components on the N atom is calculated to be 79%, which compares well with the experimental value 72%^30^. The band structures of on-center N_S_ and N_I_ are shown in Fig. 3c and 3e, which are similar to that of off-center Ns. The difference is that, the IBs of on-center N_S_ and N_I_ are nearer to the conduction band than that of off-center N_S_, but they are still separated from the conduction band. In contrast, for the N_B_ defect, the IB has merged with the valence band, as shown in Fig. 3f. Another character of these band structures is that the IBs introduced by nitrogen are half-filled, different from those introduced by the other dopants such as Ti and chalcogens.

It is known that the PBE functional underestimates the band gap of silicon. To obtain the electronic band structure with correct gap, other method and functional are used for silicon, e.g., the many-body perturbation theory based on the GW approximation^31^,^32^ and the hybrid functional proposed by Heyd *et al.* (HSE06)^26^,^27^. Previous works have shown that the HSE06 functional can provide accurate band structure for hyperdoped silicon^13^,^33^. Therefore, here we also use HSE06 to calculate the band structures for the nitrogen defects, and the results show that the calculations of PBE are qualitatively reliable. In Fig. 3b, we give the band structures of off-center N_S_ calculated from HSE06. The band gap of the off-center N_S_ is corrected to be 1.20 eV from the PBE result 0.61 eV. The impurity bandwidth of this defect is 0.114 eV in the HSE06 result, slightly different from the 0.118 eV in the PBE result. For the band structure of on-center N_S_, which is shown in Fig. 3d, the corrections of HSE06 are similar to those of the off-center one. The HSE06 results show that the IBs of off- and on-center N_S_ are still separated from the valence and conduction bands, just as the PBE results show. Moreover, the positions of the IBs relative to the Fermi level in HSE06 and PBE results are in good agreement to each other.

**Figure 4:**
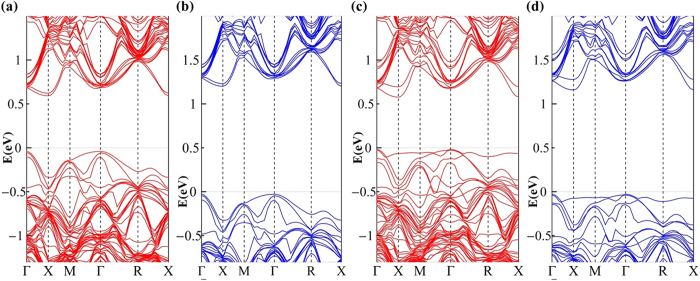
Band structures of paired nitrogen defects calculated from the PBE and HSE06 functionals. (**a**) N_I_-N_I_ (PBE), (**b**) N_I_-N_I_ (HSE06), (**c**) N_I_-N_I_-I (PBE), and (**d**) N_I_-N_I_-I (HSE06).

In Fig. 4a,c, we give the band structures of paired nitrogen defects calculated by the PBE functional. Their band structures are different from those of single nitrogen defects, there is no intermediate band in the gap. As shown in Fig. 4a, the band structure of N_I_-N_I_ is similar to that of pure silicon. For Humble ring, N_I_-N_S,_ N_S_-N_S_, N_S_-N_S-_V and N_I_-N_I_-I, our calculations show that their band structures are just slightly modified by the doped nitrogen. For instance, we present the band structure of N_I_-N_I_-I in Fig. 4c. The defect still does not introduce intermediate band into the gap. Also, we performed the HSE06 calculations for the band structures of paired defects N_I_-N_I_ and N_I_-N_I_-I respectively. As shown in Fig. 4b,d, both the band gaps are corrected to be around 1.20 eV, and the IBs do not appear in the band gaps, just as the PBE calculations show.

### Optical absorption of nitrogen hyperdoped silicon

**Figure 5:**
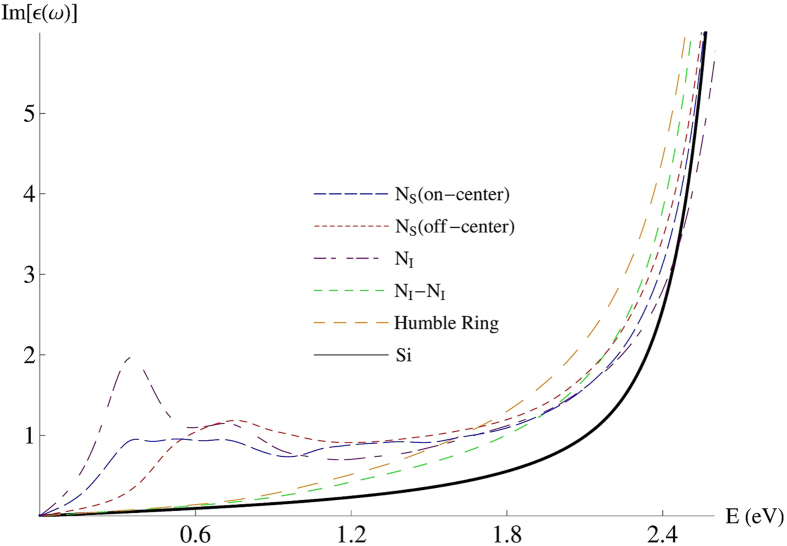
Imaginary part of the dielectric function of some representative structures for nitrogen hyperdoped silicon. The Si line represents that of pure silicon for comparison.

From the band structures as shown above, we can see that most of single nitrogen defects introduce isolated IBs deep into the band gap while paired nitrogen defects do not. It is known that the introduction of IB could bring various new optical and electrical properties for silicon. To investigate the optical properties of the nitrogen hyperdoped silicon, we calculated the dielectric function of these defect structures. In the above, by comparing with the HSE06 results, we see that the band structures calculated by PBE functional are reliable and in good qualitative agreement with those calculated by HSE06 functional. Therefore, considering the HSE06 method is rather computer-time consuming and yields not really to new physical insight into optical property, we employ PBE functional to do the optics calculations, and the results are presented in Fig. 5. As expected, paired nitrogen defects such as paired interstitial structure NI-NI, which introduce none of the impurity level into the gap, behave similar optical properties to the pure silicon and bring no infrared response. Whereas those with the IBs in the gap, i.e., N_S_ (both off- and on-center) and interstitial defects, bring sub-band-gap absorption as shown in Fig. 5. However, it is well-known that interstitial nitrogen is unstable due to the high diffusion constant as mentioned above, and can be paired or combined with vacancy within a very short time^24^,^29^, which means that the concentration of interstitial defects is negligible. Therefore, we can conclude that the sub-band-gap absorption observed in our experiments is resulted from the single nitrogen defects. More specifically, the substitutional defects including on- and off-center Ns are the origin of the sub-band-gap absorption of nitrogen hyperdoped silicon.

In sulfur hyperdoped silicon, substitutional defect is also the main source of sub-band-gap absorption. By comparing the imaginary part of dielectric function of N_S_ with that of substitutional sulfur defect from reference [34], we can see that the case of N_S_ is visibly lower than that of substitutional sulfur defect in the sub-band-gap range, which is in accordance with the experiments that nitrogen hyperdoped silicon has a lower absorptance in the near-infrared range than that of sulfur hyperdoped silicon. While, the similar mid-infrared absorptance of both hyperdoped samples observed in experiment implies there may exist other mechanism for the strong mid-infrared absorption. Finally, as mentioned above, the binding energy of the nitrogen with vacancy is as high as around 3.4 eV in N_S_ structure, which means that this structure is stable even at the melting temperature. In other words, as long as N_S_ is formed, it will keep stable even after annealing. Accordingly, the good thermal stability of the sub-band-gap absorptance observed in our experiments is related to the stability of N_S_.

## Discussion

In the past decades, the dominant nitrogen defect in silicon was thought to be N_I_-N_I_^22^, and N_S_ was rarely reported to have a considerable fraction. In fact, the formation energy of N_I_-N_I_ is small relative to that of other defects especially those with single nitrogen, as shown in Table 1. This means that in the equilibrium state, nitrogens tend to form the N_I_-N_I_ structure and the N_I_-N_I_ has an overwhelming fraction, while N_S_ is negligible. In the general doping methods, such as Czochralski-grown process in nitrogen ambient, nitrogen is doped in a near-equilibrium process. Therefore, it is easy to understand the dominance of N_I_-N_I_ and the rare report of N_S_ fraction. That is, as expected, in the near-equilibrium process the formation energy is the main factor determining the specific configuration of the dominant defect. But in a state far from equilibrium, the case may be different because dynamics factor could play a large role. As a result, the defect structures including single and cluster with higher formation energy could appear, it is then reasonable to expect the increase of N_S_ fraction. In fact, by doping nitrogen in a non-equilibrium process, ion implantation followed by nanosecond pulse laser annealing, Brower found a fraction of N_S_ to be about 10%^30^,^35^. The similar results are also found in the sulfur and selenium hyperdoped silicon respectively^9^,^12^. For the S-hyperdoped sample, although the single substitutional sulfur have higher formation energy than the paired one^36^, the single substitutional defect has a fraction between 20% to 70%^12^. For Se-hyperdoped sample, such fraction is even up to 75%^9^. In the above two cases, the preparation method and doping element are all different, but the samples all experience a highly non-equilibrium process, i.e., the melting and rapid resolidification by laser irradiation. In fact, it is already known that the pulsed-laser melting followed rapid resolidification will cause solute trapping and result in high substitutional supersaturations of dopants in silicon^37^,^38^. Therefore, in the present femtosecond-laser-hyperdoped silicon, it is reasonably deduced that N_S_ structure should occupy a considerable fraction and according to our calculations induce sub-band-gap absorption. In our previous works, the infrared spectra of nitrogen hyperdoped sample indeed shows the existence of N_S_^19^. Certainly, the specific value of N_S_ fraction needs to be further investigated.

Owing to the highly non-equilibrium process, whether the fractions of various impurity clusters, e.g, trimer and tetramer etc., also increases as those of the single substitutional defect? In fact, one has tried to find the impurity clusters in the hyperdoped silicon. Out of expectation, however, no impurity clusters have been detected in experiments so far^8^,^39^. In reference [8], the sample is S-hyperdoped silicon fabricated by ion implantation and pulsed-laser-melting-induced rapid solidification. In Cross-sectional TEM examination, there is no evidence for the presence of S clusters^8^. Recently, Newman *et al.* applied extended X-ray absorption fine structure spectroscopy to probe the chemical state of dopants in Se-hyperdoped silicon prepared by femtosecond-laser irradiation, The results show that even Se dimer is not likely present in large amounts^39^. Therefore, for hyperdoped silicon, although the impurity concentration is extremely high, the multi-dopant defect can be neglected comparing with the isolated one, the dynamics mechanisms for which are unclear now.

**Figure 6:**
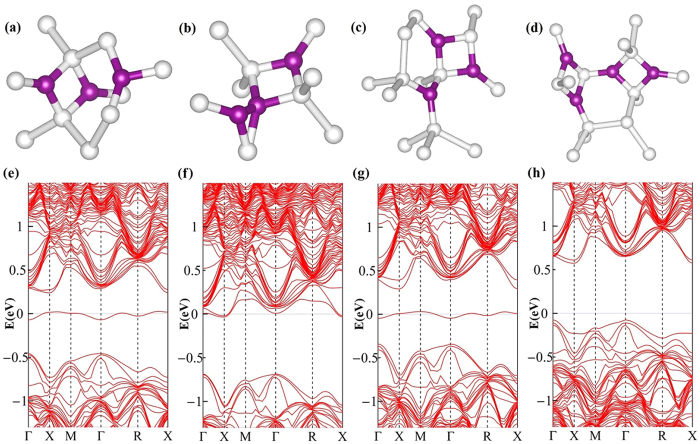
Geometries and band structures of nitrogen clusters. (**a**)-(**d**) represent the geometries of 3-N_I_(3), 3-N_I_(4), N_I_-N_I_-N_s_ and 4-N_I_ respectively, white and purple balls represent the Si and N atoms, respectively. (**e**)-(**h**) show the PBE band structures of 3-N_I_(3), 3-N_I_(4), N_I_-N_I_-N_s_ and 4-N_I_ respectively.

After the melting and rapid resolidification, however, the clusters larger than N_I_-N_I_ dimer are possible to be formed in the equilibrium stage because of the high diffusion constant of the interstitial nitrogen. Therefore, we consider the trimer and tetramer defects. For the interstitial trimer N_I_-N_I_-N_I_ (abbreviated as 3-N_I_), after the structure search and optimization, several different trimer configurations are obtained, their formation energies relative to that of N_I_-N_I_ are given in Table 2. We see that the structure with lower energy, e.g., 3N_I_(1), 3N_I_(2), and 3N_I_(3) in Table 2 is more like a combination of one N_I_-N_I_ dimer and a single interstitial N defect (N_I_ or N_B_). For example, as shown in Fig. 6a, the N-Si bond-lengths of 3N_I_(3) are within a difference of 1%, in comparing with that of isolated N_I_-N_I_ and isolated N_I_. Certainly, the cluster where nitrogens are aggregated tightly is also found as 3N_I_(4) shown in Fig. 6b for instance. But its formation energy 3.65 eV is so high that it can be neglected in equilibrium state. The above results show that the preferred interstitial trimers are a kind of combination of N_I_-N_I_ dimer and single interstitial N. For other types of cluster, e.g., N_I_-N_I_-N_s_, and tetramer N_I_-N_I_-N_I_-N_I_ (abbreviated as 4-N_I_), the cases are similar as shown in Fig. 6c,d, in which the preferred structures with lower formation energies are given. Then, for these clusters, we calculate their band structures as shown in Fig. 6e–h. The interesting result is that, for the preferred defect configurations, i.e. 3N_I_(1), 3N_I_(2), 3N_I_(3), N_I_-N_I_-N_s_, and 4-N_I_, their band structures can also be simply regarded as a kind of “combination” of those of N_I_-N_I_ dimer and isolated nitrogen defect. For example, the cluster 3N_I_(3) is a combination of N_I_-N_I_ dimer and single N_I_ as mentioned above, Then, owing to the IB present in the band structures of N_l_ while absent in that of N_I_-N_I_ dimer, there also exists an IB in the band structure of 3N_I_(3) as shown in Fig. 6e, and the IB position is almost the same as that of N_l_. The case is the same for N_I_-N_I_-N_s_ cluster, whose IB characteristics is almost the same as that of single defect N_s_, as shown in Fig. 6g. For 4-N_I_ cluster consisting of two N_I_-N_I_ dimers, however, there is no IB in the gap because of the band characteristics of N_I_-N_I_ dimer, as shown in Fig. 6h. Therefore, in N-hyperdoped silicon, one can consider the clustering of the isolated defects. But the above results including the characteristics of atomic geometries and electronic structures all suggest that the N clusters can be described properly with the model of dimer and monomer. In other words, the properties of the N-hyperdoped silicon can be well described just in terms of monomer and dimer.

## Conclusions

For nitrogen hyperdoped silicon recently prepared in our group, we employed first-principles calculations to clarify the physics underlying the unique properties, such as sub-band-gap absorption and good thermal stability. We consider twelve structures relative to single and paired nitrogen defects, of which the formation energy, electronic structure, and dielectric function are given. The band structures and dielectric functions show that, different from paired nitrogen defects, single nitrogen defects can introduce IB and lead to the sub-band-gap absorption. Considering the instability of interstitial nitrogen, we conclude that the substitutional defects including on- and off-center ones are the source of sub-band-gap absorption observed in experiment. Moreover, if noticing the highly non-equilibrium femtosecond-laser preparation process, the substitutional defects should have considerable fraction in our hyperdoped sample, and then lead to the visible sub-band-gap absorption. But, in the mid-infrared range, there may exist other mechanism for the strong absorption. Finally, the stability of sub-band-gap absorption is related to the stable substitutional structure, which has large binding energy. These theoretical calculations provide the fundamental aspects for band structure engineering and application of such hyperdoped silicon.

## Methods

The supercell we used to model the defects consists of a 3 × 3 × 3 supercell of the conventional Si_8_ cubic cell. The dopant concentrations are around 2.37 × 10^20^ cm^−3^ and 4.73 × 10^20^ cm^−3^ for single and paired nitrogen defects respectively, which are comparable with the dopant concentration ~10^20^ cm^−3^ in experiment.

The calculations were based on the density functional theory (DFT) implemented in the Vienna *Ab Initio* Simulation Package (VASP)^40^,^41^. For structure optimizations and static energy calculations, we used the generalized gradient approximation (GGA) with the PBE functional^25^. 3 × 3 × 3 Monkhorst-Pack grid of reciprocal lattice points was used to sample the irreducible Brillouin zone. Every structure was relaxed to a residual force tolerance of 0.01 eV/Å on each atom, and converged results for lattice constant and bond-length are both with errors of 10^−4 ^Å. The lattice constant for crystal silicon determined with these parameters is 5.46 Å, which compares well to the experimental value of 5.43 Å. For static energy calculations, converged results of the total energy is with errors of 10^−3^ eV. The formation energies of defects were calculated by equation:


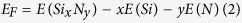


where E(Si), E(N) are chemical potentials of Si and N atom in the supercell respectively, *i.e.*, E(Si) = E(Si_216_)/216 and E(N) = [E(Si_216_ N_2_)-E(Si_216_)]/2.

For the band structure calculations, the PBE and HSE06 functionals were used respectively. In HSE06, the exact exchange is separated into a long-range part, which is essentially described by PBE, and a short range part, which is mixed with the Hartree–Fock and PBE exchanges. In our calculations, the screening parameter of μ = 0.2 is used, and the chosen mixing coefficient is 25%, i.e., 25% Hartree–Fock exchange and 75% PBE exchange. The silicon band gap obtained by the PBE functional is 0.62 eV, and it is corrected to be 1.20 eV by the HSE06 method, which compares well with the experiment value.

Finally, the optical absorption is obtained from the dielectric function. The imaginary part of dielectric function is obtained by summing over independent transitions between Kohn-Sham states neglecting local field effects^42^. 5 × 5 × 5 Monkhorst-Pack sampling of the Brillouin zone is needed for the convergence of the optical calculations.

## Additional Information

**How to cite this article**: Zhu, Z. *et al*. Electronic Band Structure and Sub-band-gap Absorption of Nitrogen Hyperdoped Silicon. *Sci. Rep.* **5**, 10513; doi: 10.1038/srep10513 (2015).

## Figures and Tables

**Table 1:** Relative formation energies per N atom for the single and pair defect structures.

**Structure**	**E (eV)**
N_T_	4.89
N_H_	4.26
N_B_	2.19
N_S_ (on-center)	2.15
N_S_ (off-center)	2.10
N_I_	1.89
N_S_-N_S_-V	1.52
N_I_-N_S_	1.13
N_I_-N_I_-I	1.05
Humble ring	0.94
N_S_-N_S_	0.74
N_I_-N_I_	0

**Table 2:** Relative formation energies for the cluster defects.

**Structure**	**E (eV)**
3-N_I_(4)	3.65
3-N_I_(3)	1.90
3-N_I_(2)	1.70
3-N_I_(1)	1.59
N_I_-N_I_-N_S_	1.15
N_I_-N_I_	0
4-N_I_	−0.51
